# Supplementary material for: Modeling glioblastoma heterogeneity as a dynamic network of cell states
Source: Mol Syst Biol. 2021 Sep 16;17(9):e10105. doi: 10.15252/msb.202010105 (PMC8444284; doi:10.15252/msb.202010105)
Supplement: Supplementary file 6 — Source Data for Figure 5 [file MSB-17-e10105-s004.zip › Figure5A_sourcedata/GSEA_3071/hallmarks_stateV.GseaPreranked.1623742546329/HALLMARK_KRAS_SIGNALING_DN.html]

Details for gene set HALLMARK\_KRAS\_SIGNALING\_DN[GSEA]

|  || Dataset | state43071 |
| Phenotype | NoPhenotypeAvailable |
| Upregulated in class | na\_pos |
| GeneSet | HALLMARK\_KRAS\_SIGNALING\_DN |
| Enrichment Score (ES) | 0.3552596 |
| Normalized Enrichment Score (NES) | 1.4889559 |
| Nominal p-value | 0.054195803 |
| FDR q-value | 0.06216775 |
| FWER p-Value | 0.69 |
Table: GSEA Results Summary

  

Fig 1: Enrichment plot: HALLMARK\_KRAS\_SIGNALING\_DN      
 Profile of the Running ES Score & Positions of GeneSet Members on the Rank Ordered List

  

| PROBE | GENE SYMBOL | GENE\_TITLE | RANK IN GENE LIST | RANK METRIC SCORE | RUNNING ES | CORE ENRICHMENT || 1 | IGFBP2 |  |  | 8 | 1.922 | 0.0873 | Yes |
| 2 | BARD1 |  |  | 132 | 1.310 | 0.1244 | Yes |
| 3 | KLHDC8A |  |  | 234 | 1.134 | 0.1576 | Yes |
| 4 | KCNQ2 |  |  | 270 | 1.091 | 0.2013 | Yes |
| 5 | GAMT |  |  | 327 | 1.040 | 0.2387 | Yes |
| 6 | YPEL1 |  |  | 398 | 0.951 | 0.2694 | Yes |
| 7 | CELSR2 |  |  | 431 | 0.923 | 0.3060 | Yes |
| 8 | CNTFR |  |  | 598 | 0.801 | 0.3114 | Yes |
| 9 | TFAP2B |  |  | 884 | 0.666 | 0.2881 | Yes |
| 10 | SHOX2 |  |  | 1128 | 0.586 | 0.2689 | Yes |
| 11 | MSH5 |  |  | 1136 | 0.582 | 0.2945 | Yes |
| 12 | SNN |  |  | 1360 | 0.517 | 0.2760 | Yes |
| 13 | EFHD1 |  |  | 1372 | 0.514 | 0.2976 | Yes |
| 14 | C5 |  |  | 1392 | 0.508 | 0.3175 | Yes |
| 15 | CCDC106 |  |  | 1449 | 0.492 | 0.3296 | Yes |
| 16 | DCC |  |  | 1504 | 0.479 | 0.3415 | Yes |
| 17 | SLC16A7 |  |  | 1547 | 0.471 | 0.3553 | Yes |
| 18 | PAX3 |  |  | 1819 | 0.415 | 0.3229 | No |
| 19 | TGFB2 |  |  | 1976 | 0.392 | 0.3114 | No |
| 20 | MFSD6 |  |  | 2135 | 0.370 | 0.2985 | No |
| 21 | SLC25A23 |  |  | 2334 | 0.344 | 0.2767 | No |
| 22 | PTPRJ |  |  | 2395 | 0.335 | 0.2808 | No |
| 23 | CLSTN3 |  |  | 2834 | 0.285 | 0.2108 | No |
| 24 | CHST2 |  |  | 3126 | 0.258 | 0.1674 | No |
| 25 | SLC38A3 |  |  | 3170 | 0.253 | 0.1710 | No |
| 26 | PLAG1 |  |  | 3228 | -0.255 | 0.1719 | No |
| 27 | LFNG |  |  | 3749 | -0.339 | 0.0888 | No |
| 28 | IFI44L |  |  | 4387 | -0.497 | -0.0092 | No |
| 29 | GPRC5C |  |  | 4890 | -0.801 | -0.0676 | No |
| 30 | EDN1 |  |  | 5038 | -1.014 | -0.0486 | No |
| 31 | TENM2 |  |  | 5047 | -1.028 | -0.0027 | No |
| 32 | COPZ2 |  |  | 5069 | -1.073 | 0.0429 | No |
Table: GSEA details [plain text format]

  

Fig 2: HALLMARK\_KRAS\_SIGNALING\_DN: Random ES distribution      
 Gene set null distribution of ES for **HALLMARK\_KRAS\_SIGNALING\_DN**
